# Reactive Oxygen Species and Mitochondrial Dynamics: The Yin and Yang of Mitochondrial Dysfunction and Cancer Progression

**DOI:** 10.3390/antiox7010013

**Published:** 2018-01-16

**Authors:** Jan Ježek, Katrina F. Cooper, Randy Strich

**Affiliations:** Department of Molecular Biology, Rowan University Graduate School of Biomedical Sciences, Stratford, NJ 08084, USA; jezekj6@rowan.edu (J.J.); strichra@rowan.edu (R.S.)

**Keywords:** mitochondrial dynamics, mitochondrial ultrastructure, redox signaling, oxidative stress, reactive oxygen species, superoxide, cancer

## Abstract

Mitochondria are organelles with a highly dynamic ultrastructure maintained by a delicate equilibrium between its fission and fusion rates. Understanding the factors influencing this balance is important as perturbations to mitochondrial dynamics can result in pathological states. As a terminal site of nutrient oxidation for the cell, mitochondrial powerhouses harness energy in the form of ATP in a process driven by the electron transport chain. Contemporaneously, electrons translocated within the electron transport chain undergo spontaneous side reactions with oxygen, giving rise to superoxide and a variety of other downstream reactive oxygen species (ROS). Mitochondrially-derived ROS can mediate redox signaling or, in excess, cause cell injury and even cell death. Recent evidence suggests that mitochondrial ultrastructure is tightly coupled to ROS generation depending on the physiological status of the cell. Yet, the mechanism by which changes in mitochondrial shape modulate mitochondrial function and redox homeostasis is less clear. Aberrant mitochondrial morphology may lead to enhanced ROS formation, which, in turn, may deteriorate mitochondrial health and further exacerbate oxidative stress in a self-perpetuating vicious cycle. Here, we review the latest findings on the intricate relationship between mitochondrial dynamics and ROS production, focusing mainly on its role in malignant disease.

## 1. Introduction

### 1.1. From Bean to Interconnected Web

In textbooks, mitochondria are classically depicted as round, bean-like organelles [1]. The oval shape of mitochondria observed by electron microscopy is consistent with the endosymbiotic theory that mitochondria evolved from a bacterial ancestor [2,3,4]. However, in living cells, mitochondria typically display dynamic networks of interconnected tubules with many branching points (Movie S1). The fluctuating nature of mitochondrial ultrastructure affords these organelles flexibility in regulating the bioenergetic flux of key molecular elements such as ATP, lipids, proteins, mitochondrial DNA (mtDNA), metabolites, cofactors, and ions throughout the entire mitochondrial network [5,6]. Small guanosine 5′-triphosphatase (GTPase) proteins like Miro help provide a high degree of homeostatic heterogeneity within the mitochondrial matrix by trafficking mitochondria along cytoskeletal filaments within [7,8] or between cells [9], thereby exposing mitochondria to distinct biochemical milieus [10]. Although mitochondrial motility was predominantly studied in neurons where mitochondria serve a specialized function [11], recent literature has revealed molecular players of the neuronal mitochondrial transport machinery that may be deregulated in cancers [12]. In this scenario, these molecular motors hijack mitochondria and transfer them into cancer cell peripheries as a localized energy supply for increased mobility and metastasis of the tumor. Accordingly, mitochondrial fragmentation is believed to facilitate repositioning of mitochondria in cancer cells [13].

### 1.2. The Fission/Fusion Machinery

The overall steady-state morphological appearance of the mitochondrial reticulum is governed by the opposing activities of large GTPases directing either fission or fusion. Fusion of the outer and inner mitochondrial membrane (OMM and IMM) is mediated by mitofusins Mfn1 [14] or Mfn2 [15] and optic atrophy 1 (Opa1) [16,17], respectively. Profusion activity has also been reported for mitochondrial phospholipase D (PLD) [18] and mitofilin [19], which is part of the newly-discovered mitochondrial contact site and cristae organizing system (MICOS) [20,21]. In response to oxidative stress [22,23,24] or during cell division [25], the recruitment and activation of the mitochondrial fission GTPase, dynamin-related protein 1 (Drp1), shifts the balance toward the fragmented phenotype [26,27,28]. Oligomerization of Drp1 into a spiral-shaped filament around a mitochondrial tubule precedes the simultaneous constriction of both OMM and IMM, which occurs in a GTP-dependent manner [29]. Mitochondrial fission factor (Mff), mitochondrial fission 1 (Fis1), or mitochondrial dynamic proteins 49 and 51 (MiD49 and MiD51) are receptor-adapters that actively facilitate Drp1 interaction with the OMM during mitochondrial division [30,31,32]. Drp1 is post-translationally regulated via phosphorylation, ubiquitination, SUMOylation, and *S*-nitrosylation. For example, phosphorylation at Ser616 by the protein kinase C isoform δ (PKCδ) is activating, whereas phosphorylation at Ser637 by protein kinase A (PKA) inhibits Drp1 function [33]. Moreover, activity of the classic GTPase dynamin 2 (Dyn2) is required for final mitochondrial scission, indicating that this process is complex, requiring multiple steps and contributing factors [34].

### 1.3. Linking Mitochondrial Dynamics to Cell Death Pathways

**Figure 1:**
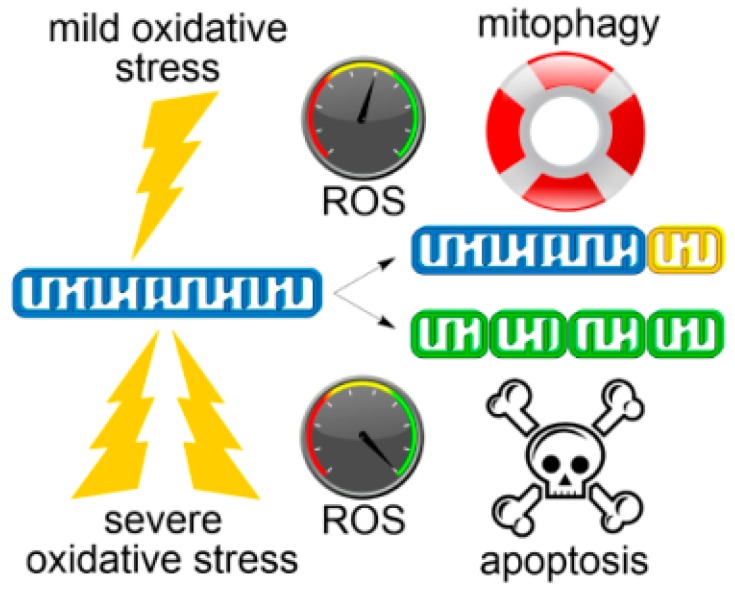
Changing cell fates depending on oxidative damage extent and mitochondrial dynamics. Under mild oxidative stress conditions (top), clearance of defective mitochondrion (yellow) by mitophagy reduces ROS levels and enhances cell survival. Acute oxidative stress (bottom) promotes extensive mitochondrial fission and dysfunction that ultimately leads to elevated ROS, loss of mitochondrial integrity (green) and apoptotic cell death.

Fragmentation of the mitochondrial network is linked with several physiological indicators of mitochondrial dysfunction, including loss of mitochondrial membrane potential (∆*Ψ*_m_), decreased respiration and oxidative phosphorylation (OXPHOS), metabolic shift towards glycolysis, and increased mitochondrial ROS formation [33]. Glucose or fatty acid catabolism may also give rise to fragmented mitochondria, albeit with hyperpolarized ∆*Ψ*_m_ and augmented OXPHOS [35]. Collapse of the reticular form of mitochondria into fragments represents a key intermediate accompanying the activation of either salvaging or pro-death pathways depending on the stimulus. Resultant fragments of dysfunctional mitochondria can be selectively targeted for mitophagy to promote cell survival [36] or, in severe oxidative stress, trigger the intrinsic pathway of apoptosis (Figure 1) [37]. A common denominator for apoptotic and mitophagic stress response is the involvement of mitochondrial fission, perhaps triggered by mitochondrial ROS [38,39].

Intrinsic, or mitochondrial, apoptosis is characterized by increased association of the pro-apoptotic protein Bcl-2-associated X (Bax) with the OMM [40]. Bax, and its close homolog Bcl-2 homologous antagonist/killer (Bak), which constitutively resides in OMM, oligomerize to induce mitochondrial outer membrane permeabilization (MOMP). MOMP is accompanied by the release of pro-apoptotic factors, including cytochrome C, apoptosis-inducing factor (AIF), second mitochondria-derived activator of caspase/direct inhibitor of apoptosis-binding protein with low pI (Smac/DIABLO), Omi/high temperature requirement protein A2 (Omi/HtrA2), and other second-messengers that initiate either caspase-dependent or caspase-independent cell death [41,42]. Although regulation of MOMP by mitochondrial dynamics is still elusive, considerable progress has been made towards visualizing Bax-mediated OMM pore formation in cells by means of super-resolution microscopy [43,44,45]. Consistent with a positive role for fission in programmed cell death (PCD), promoting fusion or inhibiting fragmentation prevents or delays the onset of apoptosis [46] or mitophagy [47]. Given that evading apoptosis is a hallmark of cancer, correct control of mitochondrial dynamics is critical for slowing or inhibiting tumor progression [22,36,48,49,50]. In addition, failure of these mitochondrial quality control check-points can contribute to the development of degenerative pathologies such as type 2 diabetes [51,52,53], heart and brain ischemia-reperfusion (I/R) injury [54], cardiovascular disorders [55,56,57,58,59], and neuropathies [59,60,61] such as Parkinson’s [62] and Alzheimer’s disease [63]. Mitochondrial dysregulation is also thought to contribute to physiological processes associated with aging [64,65]. Are fragmented mitochondria a cause or consequence of ROS overproduction? Is it the severity of mitochondrial dysfunction that discriminates between redox signaling and oxidative stress? Can oxidative stress be regulated by mitochondrial morphology? Are there different mitochondrial fission phenotypes? Can we therapeutically treat mitochondrial dynamics? This review focuses on the interplay between mitochondrial dynamics and ROS homeostasis relevant to the etiology of malignant neoplastic diseases.

## 2. Modulation of Mitochondrial Dynamics by Reactive Oxygen Species

### 2.1. Mitochondria Are a Prime Source of ROS

A primary function of mitochondria is to generate proton-motive force, required for ATP synthesis during OXPHOS to support cellular processes. As such, mitochondria constitute a major metabolic hub, or a “molecular furnace”, in which catabolic pathways intersect to oxidize nutrients. Along the electron transport chain (ETC), up to 2% of electrons leak before reaching Complex IV, incompletely reacting with oxygen in a one-electron reduction to produce superoxide (anion radical) instead of a water molecule [66]. Mitochondria are the major producer of superoxide and other downstream ROS in the cell [67], the main source of mitochondrially-derived superoxide being Complex I and III [68]. Additionally, mitochondrial superoxide is generated by electron-transferring flavoprotein-ubiquinone oxidoreductase during fatty acid oxidation, glycerol-3-phosphate dehydrogenase, and other IMM-associated oxidoreductases [69]. Superoxide is membrane-impermeant and is readily dismutated to non-radical, membrane-diffusible, H_2_O_2_ by superoxide dismutase (SOD) or non-enzymatic mechanisms [70,71]. As a membrane-diffusible species, mitochondrially-generated H_2_O_2_ has been implicated in cell-wide redox signal transduction [72,73,74,75,76,77]. H_2_O_2_ can be further reduced by divalent metal ions or superoxide in the Fenton [78] or Haber-Weiss reactions [79], respectively, to produce the hydroxyl radical (^●^OH). This highly toxic molecule promiscuously reacts with a broad range of metabolites, thus inducing oxidative damage [80]. Depending on the physiological context, NADPH oxidases (NOX) and dual oxidases (DUOX) can further contribute to cellular superoxide formation [81].

### 2.2. Mitochondrial ROS and Cancer, a Double-Edged Sword

ROS are well known activators of apoptosis [82,83]. Due to oncogenic mutations, increased metabolic activity, or decreased mitochondrial function, many cancer types are intrinsically associated with elevated ROS generation [84,85,86] and mitochondrial fragmentation [33,87]. In addition to being a mutagenic motor that can drive a cell out of senescence, conventional anti-cancer strategies utilize pro-oxidant drugs to push the ROS levels beyond a death-inducing threshold specifically in cancer but not normal cells [88,89,90,91]. A typical example is cisplatin, which stimulates both nuclear DNA and mtDNA damage with concomitant mitochondrial fission [22] and increased mitochondrial ROS generation [92]. As proof of concept, antioxidants interfere with the therapeutic mechanism of cisplatin [93]. In addition to chemical pro-oxidants, singlet oxygen (^1^O_2_) itself is a ROS molecule that has gained particular attention in the treatment of shallow tumors such as skin melanoma by photodynamic therapy (PDT). In PDT, ^1^O_2_ and free radicals are generated by irradiation of a pre-administered photosensitizing drug to induce apoptotic or necrotic cell death in cancer cells [94,95].

Dwelling in close proximity to the epicenter of oxidative metabolism, mtDNA is considered particularly vulnerable to free radicals and oxidative damage. The free radical theory of ageing is defined by the positive correlation between accumulated ROS and mtDNA mutations [96]. Considering that the mitochondrial genome encodes subunits of four out of five ETC Complexes (Complex I and III–V), mutations in mtDNA predispose ageing cells to mitochondrial dysfunction in a self-perpetuating vicious cycle [97]. Intramitochondrial mixing by coordinated mitochondrial fission and fusion cycles could represent an important complementary mechanism to preserve mtDNA integrity against oxidative injury [98]. Taken together, observations that ageing is associated with the fragmented mitochondrial phenotype and mtDNA damage would seem to suggest mitochondrial dynamics and ROS formation are mutually intertwined physiological processes that contribute to a slow decay of mitochondrial function during ageing.

### 2.3. Antioxidant Defense Systems

In order to cope with elevated ROS production, cells utilize a host of intricate antioxidant-defense mechanisms. The most abundant intracellular antioxidant, glutathione (GSH), participates in H_2_O_2_ detoxification through thiol-disulfide interchange catalyzed by glutathione peroxidase (GPX), yielding oxidized glutathione (GSSG) [99,100]. Glutathione reductase, in turn, plays a key role in maintaining the reduced form of glutathione [101]. In addition to its direct antioxidant capacity, GSSG contributes to antioxidant defense by inducing mitochondrial hyper-fusion in an Mfn1- and Mfn2-dependent manner [102]. Other H_2_O_2_ scavengers include catalase, which is mainly located in the peroxisomes where it converts H_2_O_2_ to water and oxygen [103], and the peroxiredoxin (PRX) system [104], relying upon the thioredoxin (TRX) and thioredoxin reductase catalytic cycle [105,106]. Stable peroxiredoxin 3 depletion in transformed mesothelioma cells leads to a hyper-fused mitochondrial phenotype, which is rescued by overexpression of mitochondrially-targeted or cytosolic catalase [107]. Additionally, antioxidant action is inherent in the activity of the uncoupling protein (UCP) family [108,109] and DJ-1 [110]. Ectopic overexpression of SET, an inhibitor of protein phosphatase 2A, in human embryonic kidney cancer cells increases UCP2 and UCP3 levels in parallel with Drp1- and Fis1-dependent mitochondrial fragmentation and diminished autophagic flux [111]. In the mouse model of I/R injury, DJ-1 elicits cardioprotection by preventing excessive mitochondrial fission [112]. Although antioxidant systems are recognized therapeutic targets, they are prone to transcriptional activation by oncogenes and thereby enhance tumorigenesis and chemoresistance [113,114]. 

## 3. Signaling Pathways Controlling Mitochondrial Dynamics

**Figure 2:**
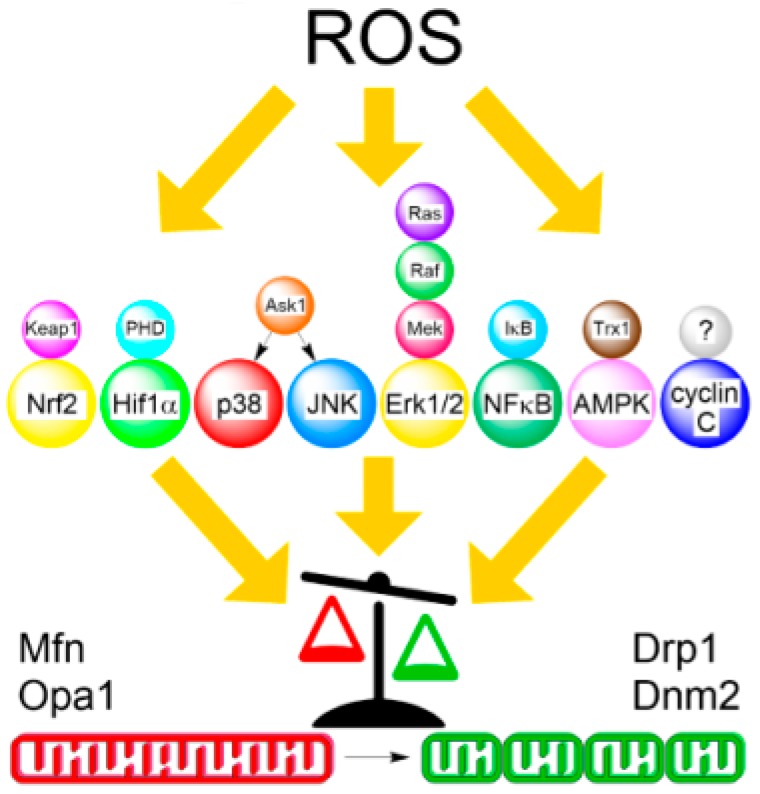
Simplified relationship between ROS-induced redox signaling and mitochondrial dynamics. ROS stimuli are recognized by redox sensors (top), which transduce the signal to their respective effector kinases (center). Phosphorylation by these kinases can either inhibit mitochondrial fusion (red) or stimulate fission (green) proteins that results in the shift of the overall balance from elongated to fragmented morphology (bottom). As indicated, Ras/ERK MAPK signaling involves multiple kinases. Redox sensor for mammalian cyclin C pathway has not yet been determined.

ROS-generating stimuli that induce mitochondrial network fragmentation can be divided into either external insults such as during treatment with pro-oxidants or chemotherapeutic agents, PDT, ionizing radiation, immune response, or viral infection that incur cell injury, or be part of physiological redox signaling, for example during hypoxia. In general, stress response signaling pathways convey both external and internal cues between a redox-sensitive receptor and the downstream effectors of the mitochondrial fission machinery (Figure 2). How these signals regulate mitochondrial fission and converge upon the apoptotic stress response is still rather enigmatic and therefore an area of active research. This section will review the current trends and paradigms of ROS-mediated signaling that form a link between redox-sensing elements and mitochondrial dynamics and their role in cancer invasiveness, tumor progression, and metastasis.

### 3.1. Nrf2/Keap1 Signaling

The master regulator of cellular stress response is the transcription factor nuclear factor (erythroid-derived 2)-like 2 (Nrf2), which reacts to exogenous stimuli by translocating from the cytosol to nucleus and initiating the expression of a broad range of antioxidant-defense and cytoprotective genes [115,116]. Since Nrf2 is upregulated in numerous tumors, it has been viewed as a promising target for cancer chemoprevention and therapy [117]. Kelch-like ECH-associated protein 1 (Keap1) is an E3 ubiquitin ligase and a redox-sensor for Nrf2 signaling that in the absence of oxidative stress targets Nrf2 for ubiquitin-mediated destruction [118]. Nrf2 is stabilized in the cytosol upon oxidative or electrophilic stress. Although Nrf2 was demonstrated to influence mitochondrial bioenergetics [119], the underlying mechanism is poorly understood. For example, mutant Huntingtin interferes with Nrf2 signaling, which resulted in increased fragmentation of mitochondrial network in response to low-level H_2_O_2_ treatment and increased susceptibility to oxidative stress in striatal cells [120]. Furthermore, Nrf2 mediates the redox-dependent effect of sulforaphane, an isothiocyanate found in cruciferous vegetables, on increased mitochondrial fragmentation, Bax induction, and the ensuing apoptosis in prostate cancer cells [121]. This response was specific to tumor cells as sulforaphane behaved as an antioxidant and promoted mitochondrial fusion and nephroprotection in parallel experiments using a non-transformed kidney cell line. Altogether, these studies imply that activation of the Nrf2 antioxidant response can be a prospective therapeutic strategy in both neurodegenerative and malignant diseases.

### 3.2. HIF1α-Mediated Response to Hypoxia

The classic example of redox sensing in cancer is the stabilization of the transcription factor hypoxia-inducible factor 1α (HIF1α) that allows solid tumors to adapt to low-oxygen stress by promoting aerobic glycolysis (Warburg’s effect), angiogenesis, and tumor invasion [122,123]. Under normoxia, hydroxylation of HIF1α by the prolyl hydroxylase domain (PHD) family of dioxygenases results in its binding to the von Hippel-Lindau protein followed by ubiquitin-dependent destruction [124]. HIF1α is stabilized in the cytosol under hypoxia due to the limited availability of oxygen for the PHD-mediated hydroxylation step. Stabilized HIF1α binds to the oxygen-insensitive subunit HIF1β and the heterodimer subsequently translocates to the nucleus to initiate the transcription of a myriad of hypoxia-response genes to promote metabolic reprogramming stimulating aerobic glycolysis while suppressing OXPHOS activity [125]. HIF1α induction stimulates expression of several glycolytic genes including phosphofructokinase, pyruvate kinase, and lactate dehydrogenase. Paradoxically, hypoxic exposure is accompanied by an initial oxidative burst of superoxide generated by mitochondria [126,127] or NOX enzymes [128]. The hypoxia-induced superoxide burst can originate from Complex I [129] or the outer Complex III ubiqinone-binding site [130]. Chandel hypothesized that the Complex III-derived superoxide signal, which is released into the mitochondrial intermembrane space, is necessary for PHD inactivation and consequent stabilization of HIF1α at the onset of hypoxia. This model implicates mitochondria as active oxygen sensors [131]. 

There is a distinct association between oxygen deprivation and mitochondrial fragmentation in malignant cells. Hypoxia-induced Drp1 overexpression and mitochondrial fission have been linked to increased migration and metastatic activity in breast cancer MDA-MB-231 [132] and U251 glioblastoma cell lines [133]. Importantly, HIF1α stabilization is required for hypoxia-induced mitochondrial fission [134]. Bioenergetic profiling identified an ovarian cell line (OVCA420) that exhibited fragmented mitochondria due to increased Drp1 expression [135]. These cells also displayed reduced respiration and increased glycolysis. In addition, OVCA420 cells failed to stabilize HIF1α in a 1% oxygen environment reducing cellular fitness to hypoxic conditions. These findings are consistent with the model that mitochondrial dynamics not only play an important role in cellular energetics, but may also serve as a sensor for the hypoxic response. Taken together, these studies suggest that HIF1α can act as a fundamental driver of both mitochondrial fission and cancer progression.

### 3.3. ASK1/p38 MAPK Pathway

The p38 mitogen-activated protein kinase (MAPK) is stimulated by the apoptosis signal-regulating kinase 1 (ASK1) protein kinase to transduce redox signals as well as other types of stress, to stimulate stress response programs, growth or differentiation depending on the signal [136,137]. Cytosolic thioredoxin 1 (Trx1) and mitochondrial thioredoxin 2 (Trx2) act as redox sensors for ASK1-mediated apoptosis signaling [138]. In their reduced form, TRXs sequester ASK1 from inducing stress- and cytokine-regulated apoptosis. This inhibition is relieved upon a pro-oxidant stimulus, which is sensed by intramolecular disulfide bond formation between Cys32 and Cys35 in TRX and this leads to the dissociation of the free kinase [139]. One role for the p38 MAPK signaling is in the adaptation of mitochondrial morphology to hypoxia. In response to hypoxia, p38 MAPK induces mitochondrial fragmentation by upregulating Siah2, which mediates derepression of Drp1 by the mitochondrial scaffolding protein AKAP121 [140]. Siah2 is an E3 ubiquitin ligase that targets AKAP121 for proteasomal degradation. In addition to p38 MAPK [141], Siah2 is upregulated by hypoxia-induced Akt signaling most likely through indirect mechanisms [142]. PKCδ-activated p38 MAPK was demonstrated to directly phosphorylate Drp1 to induce its mitochondrial translocation and subsequent mitochondrial fission in response to succinate challenge [143]. This change in mitochondrial network ultrastructure was required for promoting human mesenchymal stem cell (hMSC) motility via upregulation and redistribution of F-actin. Moreover, pharmacological inhibition of p38 MAPK prevented mitochondrial fragmentation and the onset of mitophagy in a rat brain I/R (stroke) model [144]. In addition to p38 MAPK, c-Jun N-terminal kinase (JNK) can also be activated by ASK1 in response to oxidative stress [145]. JNK has mitochondrial substrates including those important for apoptosis [146] but a role in stress-induced fission/fusion has yet to be elucidated. These data indicate that p38-driven mitochondrial fission may represent an important cytological cue to promote tumorigenesis and metastasis. 

### 3.4. Ras/ERK1/2 MAPK Pathway

The small GTPase Ras, is a well-known effector of the redox-mediated stress response [147] as well as a potent driver of malignant transformation when constitutively activated by mutation [148]. Collectively with three isoforms (Kras, Nras, and Hras), Ras is one of the most frequently mutated oncogenes occurring in approximately 25% of human tumors. When tethered to the plasma membrane through farnesyl and palmitoyl lipid moieties, Ras functions as a molecular switch oscillating between an active (GTP-bound) and inactive (GDP-bound) state. The GTP and GDP status of Ras is directed by the activity of guanine nucleotide exchange factors and GTPase-activating proteins, respectively [149,150]. Normally, Ras transduces external cues such as growth factor and cytokine stimulation to downstream MAPK cascade effectors composed of rapidly accelerated fibrosarcoma (Raf) (MAP3K), MAPK/extracellular signal-regulated kinase (MAPK/ERK) kinase 1 and 2 (MEK1/2) (MAP2K), and ERK1/2 (MAPK), to promote cell proliferation, differentiation, and motility [151]. In addition, Ras directly mediates redox signaling or initiates the oxidative stress response that can trigger apoptotic cell death [152]. Mechanistically, Ras senses ROS and RNS changes through its redox-sensitive cysteine found within a conserved consensus signature sequence NKXD, where X indicates redox-sensitive cysteine (not conserved). The reaction mechanism is thought to involve either one- or two-electron cysteine oxidation, the former being considered more common and proceeding through a thiyl radical intermediate RS^●^, where R denotes an alkyl group. RS^●^ promotes oxidation of the bound guanine nucleotide resulting in its release and rapid turnover. 

Numerous reports implicate Ras in modulating mitochondrial ultrastructure [153]. For example, Kashatus and Nascimento et al. reported that ERK2-mediated activating phosphorylation of Drp1 at Ser616 was required for mitochondrial fragmentation and tumor cell proliferation [154]. Similar effects of oncogenic Ras activation on the dynamics of mitochondria and tumor progression have been observed in cells and human melanoma cell lines [155]. In this setting, ERK1/2-dependent Drp1 activation promotes mitochondrial fission, ∆*Ψ*_m_ loss, and mitochondrial ROS generation during Ras^G12V^-driven tumor transformation. This phosphorylation mark is also used by cyclin B-Cdk1 to simulate fission at mitosis to allow the efficient partitioning of mitochondria in daughter cells [156]. Overexpression of constitutively-active Hras^G12V^ mutant or MAPK activation was associated with increased mitochondrial fission and tumor growth in both in vitro human pancreatic cancer and mouse xenograft models. This fission may be stimulating cell division by generating enough ROS to serve as both a growth catalysis and mitochondrial segregation. Drp1 depletion by shRNA-mediated knockdown or expression of phosphorylation-defective mutant prevented mitochondrial fission and Ras-induced growth. Similarly, pharmacological inhibition of the MAPK pathway led to Drp1 downregulation, increased mitochondrial fusion, and metabolic activity. These findings suggest that mitochondrial dynamics could be a viable therapeutic strategy for Ras-driven neoplasia.

### 3.5. The Canonical NF-κB Pathway

The redox-sensitive transcription factor nuclear factor κB (NF-κB) plays a major role in inflammation but is also commonly overexpressed and aberrantly activated in a variety of cancers. NF-κB activation is associated with evasion of apoptosis and tumor inflammatory or immune responses [157,158]. NF-κB is a common name for a dimeric complex formed by the members of the Rel family including p50, p52, RelA (p65), RelB and c-Rel [159]. NF-κB normally resides in the cytosol where it forms inhibitory complexes with members of the IκB protein family (IκBα, IκBβ, IκBγ, IκBε, IκBζ, p100, p105, and Bcl-3) [160,161]. Upon activation by pro-inflammatory or redox stimuli, the IκB kinase (IKK) complex composed of IKKα, IKKβ, and NF-κB essential modulator (NEMO) targets IκB proteins for ubiquitin/proteasome-mediated degradation. This frees NF-κB allowing nuclear translocation and the execution of inflammatory programs [162,163]. IKKα- and IKKαβ-deficient mouse embryonic fibroblast (MEF) cells, but not those lacking IKKβ, exhibit a fragmented mitochondrial network that positively correlate with reduced OPA1 expression [161]. In addition, both effects could be rescued by ectopic overexpression of IKKα. Curiously, PGC-1α-dependent mitochondrial biogenesis and ROS production, as well as improved mitochondrial function and antioxidant capacity, were noted in peripheral blood mononuclear cells obtained from professional football players who underwent an eight week period of active training, all of which was attributed to NF-κB activation [164].

Importantly, ROS-dependent NF-κB activation was observed in parallel with increased mitochondrial Ca^2+^ levels and mitochondrial fission in immortalized mouse kidney epithelial cells and mouse splenocytes lacking the mitochondrial tumor suppressor Fus1 [165]. Accordingly, Fus1-deficient splenocytes exerted increased ∆*Ψ*_m_ and mitochondrial ROS production. Furthermore, NF-κB and Nrf2 were implicated in cigarette smoke-induced mitochondrial fragmentation, dysfunction, and ROS generation in human airway smooth muscle (ASM) cells [166]. This was accompanied by increased Drp1 and decreased Mfn2 expression at both protein and mRNA level. In summary, these results highlight the central role of NF-κB in regulating mitochondrial structure dynamics.

### 3.6. AMPK Redox Sensing

As an energy-sensing enzyme, AMP-activated protein kinase (AMPK) is the central gatekeeper of redox and bioenergetic homeostasis [167,168,169]. Upon nutrient deprivation, AMPK induces the activation of catabolic (glycolysis, amino acid and fatty acid oxidation) and inhibition of anabolic pathways (gluconeogenesis, fatty acid biosynthesis) to restore depleted ATP levels [170]. AMPK can be allosterically activated by an increased AMP:ATP ratio or directly by low glucose levels [171]. Structurally, AMPK is a heterotrimeric protein composed of catalytic α subunit as well as regulatory β and γ subunits, each of which has distinct isoforms (two isoforms exist for subunit α and β, three for γ) [172]. AMPK is frequently downregulated during tumorigenesis, which contributes to the Warburg phenotype, tumor adaptation, and cancer progression [173]. This is consistent with a tumor suppressor function for AMPK. However, AMPK activation has been observed in a subset of tumors, which may represent a survival mechanism for poorly vascularized tumors [174,175]. Apart from being a core energy-sensor, AMPK is also implicated in redox sensing. Indeed, the presence of H_2_O_2_ [176], superoxide [177], or reactive nitrogen species (RNS), such as NO [178], can stimulate AMPK. AMPK activity is induced by Trx1-dependent reduction of cysteine residues serving as a redox regulator [179]. An alternative hypothesis claims that oxidative stress stimulates AMPK activity indirectly by elevating the AMP:ATP ratio [180]. These mechanisms are not mutually exclusive and both may contribute to AMPK regulation depending on the context.

Mitochondrial ROS levels are kept in check by peroxisome proliferator-activated receptor-gamma coactivator-1α (PGC-1α)-dependent induction of antioxidant defense mechanisms [181]. AMPK stimulates PGC-1α-dependent mitochondrial biogenesis [182], which may be important as fusion prevents mitochondrial dysfunction and ROS formation [183]. Conversely, AMPK mediates energy deprivation-induced mitochondrial fission following Complex I (rotenone) and III (antimycin A) inhibition by directly phosphorylating Mff [184]. This fragmented mitochondrial phenotype was observed following mitochondrial stress and is believed to predispose cells to mitophagy [185,186,187]. A recent study revealed a role for AMPK downstream of Drp1-mediated mitochondrial fission. Drp1 activity was augmented in brain tumor initiating (BTI) cells due to enhanced activating phosphorylation status leading to fragmented mitochondrial network morphology and increased cell survival [188]. Pharmacological inhibition or genetic knockdown of Drp1 was sufficient to induce apoptosis, which was reversed in Drp1 and AMPK double-knockdown cells, implying a modulatory role of Drp1 on AMPK function. In another report, AMPK was stimulated by the concerted activity of the oncogene Myc and an OMM phospholipase PLD6 resulting in net mitochondrial fusion [189]. The indirect, myc-driven activation of AMPK led to the repression of the transcriptional regulators Yap and Taz, whose functions are associated with tumor resistance and metastasis, resulting in poor therapeutic outcome and patient survival [190,191]. Collectively, these investigations identify AMPK as another key player in integrating oncogenic transformation events to mitochondrial morphology changes during tumorigenesis. Moreover, manipulating mitochondrial dynamics can influence basic cancer metabolism, providing a new avenue to attack this disease. 

### 3.7. Cyclin C Connects Oxidative Stress-Induced Fission to Apoptosis

Cyclin C is a highly conserved and ubiquitously expressed transcription factor that serves as a stress signaling effector that functions in the nucleus and at the mitochondria. Together with cyclin-dependent kinase 8 (Cdk8), cyclin C normally resides in the nucleus as part of the Mediator complex to regulate Cdk8 and polymerase II-dependent transcription. In the nucleus, cyclin C-Cdk8 function as co-activators with p53 to induce a subset of stress response genes [192]. Independent of transcription, cyclin C re-localizes to the mitochondria where it stimulates mitochondrial fission in response to oxidative stress in both mammalian [22] and yeast [23,24] models. In the cytoplasm, cyclin C directly activates Drp1 at OMM to facilitate mitochondrial fission. Intriguingly, oxidative stress-induced mitochondrial fragmentation did not require the Ser616 activating phosphorylation. This finding indicates that the cell recognizes stress-activated fission differently than that occurring during mitotic cell division. Furthermore, cyclin C is required for mitochondrial-dependent intrinsic, but not extrinsic, apoptosis [22]. This role for cyclin C at the mitochondria does not require Cdk8, suggesting that transcriptional regulation is not involved. In yeast cells, deletion of the mediator component *MED13* released cyclin C into the cytoplasm [193], revealing that cyclin C was necessary and sufficient to induce complete mitochondrial fragmentation. In stressed yeast cells, cyclin C release is facilitated by Med13 destruction mediated by the Skp, Cullin, F-box-containing complex (SCF) ubiquitin ligase and activating phosphorylation by the Slt2/Mpk1 MAPK [194]. Heterozygous deletion of cyclin C gene (*CCNC*) has been linked to the progression of acute lymphoblastic leukemia [195], osteosarcoma [196], and thyroid [197] cancer, suggesting that cyclin C is a bona fide tumor suppressor. Nevertheless, due to its utility, the cyclin C-mitochondrial axis is emerging as a prospective therapeutic target for both cancer and neurodegenerative diseases.

### 3.8. Other Redox Sensing Systems

Other redox signaling proteins including p53 [198], forkhead box O (FOXO) [199,200], Notch [201], PTEN [202], apurinic/apyrimidinic endonuclease 1/redox factor 1 (APE1/Ref-1) [203], ataxia-telangiectasia mutated (ATM) [204], activator protein 1 (AP-1), cAMP response element binding protein (CREB), heat shock factor 1 (HSF1), and specificity protein 1 (SP1) [205,206] have also been tangentially associated with control of mitochondrial shape and structure. How mitochondrial dynamics control by these factors is coupled to their redox sensing function remains to be clarified.

## 4. Modulation of Reactive Oxygen Species by Mitochondrial Dynamics

**Figure 3:**
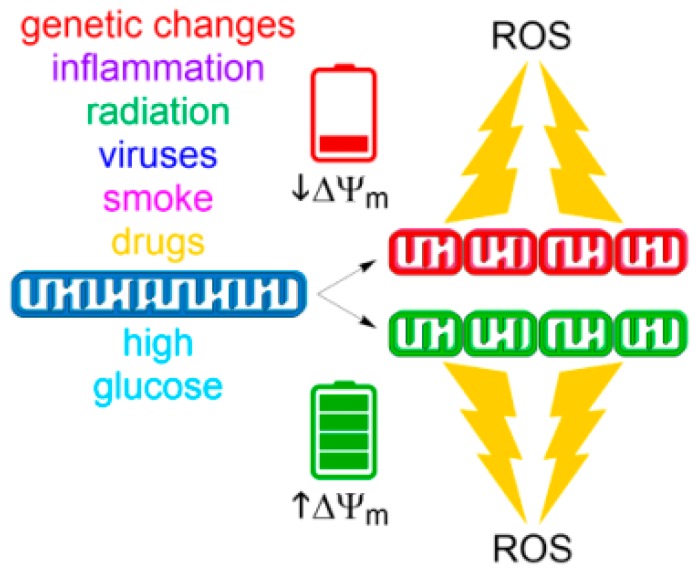
Drivers of fission-induced mitochondrial ROS production. Mitochondrial network fragmentation can occur at both extremes of ∆*Ψ*_m_. Whereas low ∆*Ψ*_m_ values result from insults causing mitochondrial dysfunction (top), high ∆*Ψ*_m_ values can be achieved by metabolic stimulation such as with high glucose (bottom). Both stimuli result in fragmented organelles and subsequent generation of superoxide by ETC.

An increasing body of evidence suggests a reciprocal link between mitochondrial morphology and ROS formation [207]. According to this hypothesis, mitochondrial shape and structure are intimately linked to the control of redox homeostasis by modulating ROS as a downstream signal (Figure 3). We will summarize recent observations of how mitochondrial fission and fusion influence mitochondrial ROS generation and how mitochondrial dynamics could be exploited as a potential therapeutic target for medical interventions.

### 4.1. Mitochondrial-Derived ROS and Fission, the Vicious Cycle

Increased mitochondrial fragmentation corresponded to an unfavorable prognosis for hepatocellular carcinoma (HCC) patients presumably due to concurrent Drp1 upregulation and downregulation of Mfn1 [208]. The fragmented mitochondrial phenotype observed after Drp1 overexpression or Mfn1 knockdown in several HCC cell lines was associated with ROS overproduction, Akt (also known as protein kinase B) activation, evasion of apoptosis, and induction of general autophagy pathway [208]. Conversely, Drp1 knockdown or Mfn1 overexpression attenuated ROS generation. Mechanistically, mitochondrial fission-induced redox activation of Akt promoted Mdm2-dependent ubiquitin-proteasomal degradation of p53 and IKK-dependent NF-κB activation [208]. Promisingly, mdivi-1, an allosteric and reversible Drp1 inhibitor, induced apoptosis and suppressed the growth of xenograft HCC tumors in immunodeficient mouse model [208]. Overexpression of Mff in human immortalized fibroblasts was reported to induce extensive mitochondrial fragmentation and concomitant mitochondrial dysfunction characterized by ∆*Ψ*_m_ loss, inefficient OXPHOS, and subsequent ATP depletion, giving rise to increased oxidative stress along with concomitant activation of mitophagy and NF-κB-dependent autophagy [209]. Consequently, Mff-overexpressing fibroblasts exhibited metabolic reprogramming towards aerobic glycolysis and excessive lactate secretion, which supported the growth of breast cancer cells in a paracrine-like manner when co-injected into nude mice. Mitochondrial dynamics in cancer-associated stromal cells could therefore be an important driver of early tumorigenesis in neighboring cancer cells as well as an eventual therapeutic target. Recent work has shed more light on the role of NF-κB-inducing kinase (NIK), a principal component of the non-canonical NF-κB pathway, in controlling mitochondrial network dynamics and subcellular localization of mitochondria in relation to tumor malignancy and invasiveness [210]. Using loss- and gain-of-function approaches, Jung and Ravi et al. have shown that NIK relies on Drp1 to mediate mitochondrial fission. This was observed in MEF cells, several cancer cell lines, as well as patient glioblastoma tissue samples. NIK recruits Drp1 to OMM, facilitates its phosphorylation-dependent activation, and consequent mitochondrial fragmentation independently of IKKα, IKKβ, and NF-κB. Consistently, intracellular ROS levels were decreased in both NIK- or Drp1-deficient glioma BT25 cells. Apart from regulating mitochondrial shape, these authors also revealed the ability of NIK to promote mitochondrial migration and motility towards cell periphery and this coincided with increased tumor invasiveness. 

Dynamic changes in mitochondrial network morphology were recognized to participate in the reprogramming of somatic cells into induced pluripotent stem cells (iPSCs) [211]. Mfn1- or Mfn2-deficient MEF cells transduced with the pluripotency factor cocktail containing Oct4, Sox2, Klf4, and c-Myc (OSKM) displayed fragmented mitochondria and increased ROS formation [212]. Such redox stimulus induced HIF1α stabilization even in the absence of hypoxia. In addition, Mfn1 or Mfn2 depletion in OSKM-treated MEF cells downregulated p53 and p21 and activated Ras and Raf. Altogether, this contributed to a genetic switch causing metabolic reprogramming from OXPHOS to glycolysis, which was identified in MEF-derived iPSCs. Analogically, mitochondrial dynamics has been implicated in the regulation of neural stem cell (NSC) maintenance and self-renewal [213]. Depletion of both Mfn1 and Mfn2 or Opa1 increased mitochondrial fragmentation and superoxide production without causing oxidative damage, consistent with the signaling role of mitochondrial fission-induced ROS. When assessed by a neurosphere formation assay, Opa1-deficient NSCs displayed compromised self-renewal capacity, which was reversed by antioxidant treatments. Conversely, Drp1-deficient NSCs showed opposite effects. In addition, employing rotenone or genetic deletion of AIF to specifically induce Complex I-mediated superoxide generation phenocopied the effect of Opa1 loss on neurosphere formation. Further supporting the role of mitochondrial dynamics in determining the fate of NSCs, mitochondrial morphology-regulated redox signaling was necessary for the execution of Nrf2-dependent developmental program responsible for the differentiation of NSCs into neurons.

### 4.2. Metabolic Stimulation

The outcome that metabolic changes have on mitochondrial function and dynamic responses can be different than those observed following genetic mutation. For example, in a cardiovascular injury model, hyperglycemic conditions trigger Drp1-mediated mitochondrial fragmentation and concomitant ROS formation that resulted in mitochondrial permeability transition (MPT)-dependent apoptosis [214]. This was observed in both rat heart myoblast H9c2 cell line and primary neonatal cardiomyocytes. In H9c2 cells, mitochondrial fission induced by high glucose levels (20 mM) stems from elevated mitochondrial metabolism and therefore cannot be associated with defective mitochondrial function [215]. In analogy, succinate induced Drp1-dependent mitochondrial fission in hMSC that stimulated ROS generation at high ∆*Ψ*_m_ and OXPHOS activity [143]. These results indicate that sufficient ROS can be generated by accelerated metabolism to push the cell past the cell death threshold. An interesting study found that in a model of diabetic nephropathy (DN), exposure of kidney glomerular mesangial cells to high glucose levels leads to mitochondrial translocation of Drp1, and subsequent mitochondrial fragmentation, increased ROS production, lipid peroxidation, p38 MAPK activation, and collagen IV synthesis [216]. Mdivi-1 abolished these effects including the translocation of Drp1 to mitochondria. Moreover, treatment with the antioxidant *N*-acetyl-l-cysteine (NAC) attenuated p38 MAPK phosphorylation, suggesting that Drp1-induced ROS activate p38 MAPK in a feed-forward regulatory loop. Inquiries into the spatial behavior of mitochondria during metabolic overload and/or reprogramming deserve further scientific attention not only in cancer but also in other metabolic disorders such as type 2 diabetes.

### 4.3. Inflammatory Stimulation

Exposure of mouse embryonic endothelial (MEE) or COS-7 cells to transforming growth factor beta (TGFβ) led to increased mitochondrial fragmentation, which positively correlated with mitochondrial superoxide overproduction [217]. Treatment with the TGFβ receptor I (TβRI) kinase inhibitor SB431542 reversed both effects in control MEE cells but not upon knockdown of the TβRI kinase substrate Smad2. Indeed, the authors went on to show that Smad2 promotes mitochondrial fusion by directly interacting with Mfn2 and recruiting the Rab and Ras interactor 1 (RIN1) as the guanine nucleotide exchange factor for Mfn2. Furthermore, an elegant study investigated the molecular underpinnings of osteogenic dysfunction during TNFα-induced inflammation [218]. TNFα treatment led to increased mitochondrial ROS formation and Drp1 expression followed by excessive mitochondrial fragmentation. Strikingly, both symptoms of mitochondrial dysfunction were inhibited by NAC or mdivi-1 treatment, thus further supporting the notion that superoxide generation and Drp1-mediated fission are intimately interlinked phenomena. Furthermore, T cell activation by cluster of differentiation 3 (CD3) antibodies has been reported to induce mitochondrial fission and ROS production in a Drp1-dependent manner [219]. The activation of Drp1 proceeded through T cell receptor (TCR)-dependent NO signaling and resulted in production of interleukin 2 (IL-2) and the CD95 via ROS-activated NF-κB. Increased levels of IL-2 and CD95 led to simultaneously augmented rates of proliferation and extrinsic apoptosis in CD3-activated T cells. 

Lipopolysaccharide (LPS), a surface glycolipid of Gram-negative bacteria and an inflammatory endotoxin, has been found to stimulate mitochondrial fission, mitochondria- and NOX-derived superoxide production in immortalized microglial cell line [220]. As a regulated event, the observed ROS formation occurred downstream of the change in mitochondrial morphology since the effects were abolished by treatment with the antioxidant oleuropein, a secoiridoid glycoside abundant in olive leaf, which inhibited Drp1 Ser637 dephosphorylation as well as LPS-induced inflammation. To summarize, mitochondrial dynamics is an essential component for the inflammatory response that directs proliferation or cell death depending on the immune signal.

### 4.4. Anticancer Drugs

In addition to endogenous metabolites and compounds, several chemotherapeutic agents are proposed to generate ROS through altered mitochondrial morphology. Inhibitor of the hedgehog signaling pathway, cyclopamine tartrate, elicited antiproliferative and pro-apoptotic behavior in non-small-cell lung cancer cell lines by inducing mitochondrial fission accompanied by decreased respiration, increased ∆*Ψ*_m_, and ROS formation [221]. Cambogin, a naturally-occurring polycyclic polyprenylated acylphloroglucinol, displayed cytostatic and apoptosis-inducing effects in breast cancer cell lines. Increased mitochondrial superoxide as well as cytosolic NADPH oxidase 1 (Nox1)-mediated ROS generation paralleled elevated mitochondrial fission and diminished ∆*Ψ*_m_ following cambogin treatment [222]. The mechanism of action of cambogin may involve the release of ASK1 from inhibition by Trx1. Activated ASK1 is then free to stimulate apoptosis via JNK signaling. Nox1 inhibition or siRNA-mediated knockdown restored normal mitochondrial morphology and ROS production to basal values. The authors concluded that mitochondrial ROS may converge upon mitochondrial dynamics downstream of Nox1 activation. This interpretation is again consistent with the feed-forward regulatory mechanism of mitochondrial ROS-induced ROS generation during structural remodeling of mitochondria. Moreover, silica nanoparticles were reported to induce mitochondrial fragmentation in human umbilical vein endothelial cells, which was caused by dysregulation of the fission machinery proteins, ∆*Ψ*_m_ loss, and accompanied by increased mitochondrial ROS formation and decreased mtDNA copy number [223]. These studies underscore the fact that mitochondrial ultrastructure changes can be inherently part of the mechanism of action of ROS-generating chemotherapeutics.

### 4.5. Ionizing Radiation

Ionizing radiation composed of α particles ($\begin{array}{c} 4\\ 2 \end{array}$He^2+^) has been documented to upregulate Drp1 expression, induce mitochondrial fission, and cause damage to Complex II and IV in immortalized human small airway epithelial (SAE) cells [224]. Elevated levels of mitochondrial superoxide were one of the characteristics associated with α particle radiation-induced mitochondrial dysfunction. Whereas mdivi-1 pretreatment preserved the integrity of mitochondrial network after exposure of SAE cells to α radiation, it did not immediately rescue mitochondrial function implying that α particles cause direct damage to ETC components. 

### 4.6. Viral Infection

Simultaneously increased ROS and mitochondrial fragmentation due to perturbed mitochondrial function were observed during the infection of astrocytoma U-87MG (glioma) cells by the vaccine strain of Venezuelan equine encephalitis virus (VEEV) [225]. Mdivi-1 treatment prevented VEEV-induced mitochondrial fission and doxorubicin-induced apoptosis in U-87MG cells. Although the exact mechanism of VEEV infection is unknown, these data confirm that viruses are capable of impacting on the fission/fusion machinery to predispose infected cells towards cell death.

### 4.7. Cigarette Smoke

Cigarette smoke can be regarded as oxidative insult [226]. Cigarette smoke-induced mitochondrial ROS production that was observed in ASM cells [166] was prevented by Drp1 siRNA-mediated knockdown. Conversely, Mfn2 knockdown elevated mitochondrial ROS levels in the presence or absence of cigarette smoke. These results further support the role of mitochondrial dynamics in modulating mitochondria-generated ROS.

## 5. Conclusions

**Figure 4:**
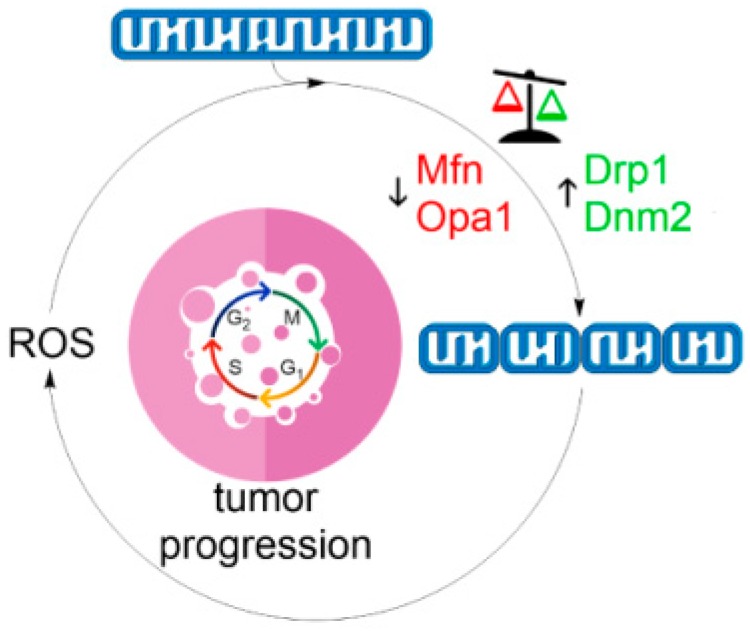
The vicious cycle of mitochondrial fragmentation and ROS production. Fission of the mitochondrial network, such as during oxidative stress (Figure 2), induces mitochondrial ROS generation (Figure 3) initiating a self-perpetuating cycle promoting mitochondrial dysfunction, cell cycle progression and genetic mutation that stimulates neoplastic growth and tumor initiation.

Mitochondrial network dynamics has important consequences for physiological homeostasis as well as in disease states. A vital determinant of mitochondrial structure is the mitochondrial membrane potential. Within physiological values of ∆*Ψ*_m_, mitochondria are interconnected and elongated. However, at both low (mitochondrial dysfunction) and high (metabolic saturation) extremes of ∆*Ψ*_m_, mitochondria display a fragmented phenotype accompanied by increased ROS generation. Given that mitochondrial fission represents an early step in apoptosis, mitochondrial ROS play a fundamental role at the intersection between these two processes. Although visually similar in appearance, two types of fragmented state should be distinguished—reversible and irreversible. Whether fragmentation of mitochondrial network commits cells to cell death is likely to be indicated by the presence of the latter phenotype. Structurally, this may be the terminal state of ROS-induced ROS vicious cycle that may involve escalating mitochondrial fission as a core component of this mechanism (Figure 4) [227]. Hence, manipulating mitochondrial morphology towards fission by pro-oxidants or towards fusion by antioxidants or mdivi-1 seems to be a viable therapeutic strategy in combating cancer and degenerative diseases, respectively [228].

## Supplementary Materials

The following are available online at www.mdpi.com/2076-3921/7/1/13/s1. Movie S1: Movie illustrates 4-D imaging of mitochondrial network dynamics in living human hepatoma HepG2 cells permanently expressing mitochondrially-targeted GFP using a Zeiss confocal microscope. The temporal axis does not correspond to a real time sequence due to the extended acquisition time required to capture each image in high resolution.
